# Spectral Detector CT-Derived Pulmonary Perfusion Maps and Pulmonary Parenchyma Characteristics for the Semiautomated Classification of Pulmonary Hypertension

**DOI:** 10.3389/fcvm.2022.835732

**Published:** 2022-02-28

**Authors:** Roman Johannes Gertz, Felix Gerhardt, Jan Robert Kröger, Rahil Shahzad, Liliana Caldeira, Jonathan Kottlors, Nils Große Hokamp, David Maintz, Stephan Rosenkranz, Alexander Christian Bunck

**Affiliations:** ^1^Department of Radiology, Faculty of Medicine and University Hospital Cologne, University of Cologne, Cologne, Germany; ^2^Department of Cardiology, Faculty of Medicine and University Hospital Cologne, University of Cologne, Cologne, Germany; ^3^Department of Radiology, Neuroradiology, and Nuclear Medicine, Johannes Wesling University Hospital, Ruhr University Bochum, Bochum, Germany; ^4^Clinical Applications Research, Philips GmbH Innovative Technologies, Aachen, Germany

**Keywords:** spectral detector CT, dual energy, pulmonary hypertension, pulmonary perfusion maps, virtual non-contrast pulmonary parenchyma characteristics

## Abstract

**Objectives:**

To evaluate the usefulness of spectral detector CT (SDCT)-derived pulmonary perfusion maps and pulmonary parenchyma characteristics for the semiautomated classification of pulmonary hypertension (PH).

**Methods:**

A total of 162 consecutive patients with right heart catheter (RHC)-proven PH of different aetiologies as defined by the current ESC/ERS guidelines who underwent CT pulmonary angiography (CTPA) on SDCT and 20 patients with an invasive rule-out of PH were included in this retrospective study. Semiautomatic lung segmentation into normal and malperfused areas based on iodine density (ID) as well as automatic, virtual non-contrast-based emphysema quantification were performed. Corresponding volumes, histogram features and the ID Skewness_PerfDef_-Emphysema-Index (δ-index) accounting for the ratio of ID distribution in malperfused lung areas and the proportion of emphysematous lung parenchyma were computed and compared between groups.

**Results:**

Patients with PH showed a significantly greater extent of malperfused lung areas as well as stronger and more homogenous perfusion defects. In group 3 and 4 patients, ID skewness revealed a significantly more homogenous ID distribution in perfusion defects than in all other subgroups. The δ-index allowed for further subclassification of subgroups 3 and 4 (*p* < 0.001), identifying patients with chronic thromboembolic PH (CTEPH, subgroup 4) with high accuracy (AUC: 0.92, 95%-CI, 0.85–0.99).

**Conclusion:**

Abnormal pulmonary perfusion in PH can be detected and quantified by semiautomated SDCT-based pulmonary perfusion maps. ID skewness in malperfused lung areas, and the δ-index allow for a classification of PH subgroups, identifying groups 3 and 4 patients with high accuracy, independent of reader expertise.

## Introduction

Pulmonary hypertension (PH) describes a rare group of diseases that are defined by an increase in mean pulmonary artery pressure (mPAP) at rest as assessed by right heart catheterization (RHC). While the current ESC/ERS guidelines define PH as an increase in the mPAP ≥ 25 mmHg (1) the 6th World Symposium on Pulmonary Hypertension Task Force more recently suggested a new pressure level of > 20 mmHg to define an abnormal elevation in the mPAP (2). According to the ESC/ERS guidelines PH is clinically classified based on hemodynamic characteristics and the review of a comprehensive set of clinical investigations. The clinical classification defines five subgroups: pulmonary arterial hypertension (PAH, group 1), PH due to left heart disease (group 2), PH due to lung disease and/or hypoxia (group 3), chronic thromboembolic PH (CTEPH) and other pulmonary artery obstructions (group 4) and PH with unclear/multifactorial mechanisms (group 5). While all groups share an increase in the mPAP, the hemodynamic classification differentiates between precapillary PH, defined by a pulmonary arterial wedge pressure (PAWP) ≤ 15 mmHg and a concomitant elevation in pulmonary vascular resistance (PVR) ≥ 3 Wood Units (WU) (group 1, 3, 4, 5) and post-capillary PH (PAWP > 15 mmHg, PVR <3 WU, group 2 and 5) (1, 2). Based on the diastolic pressure difference (DPG) group 2 is further subclassified into isolated precapillary PH (Ipc-PH, DPG <7 mmHg, PVR <3 WU) and combined pre- and postcapillary PH (Cpc-PH, DPG ≥ 7 mmHg and PVR >3 WU) (1). The underlying pathophysiological mechanisms that lead to loss and obstructive remodeling of the pulmonary vascular bed are diverse and remain incompletely understood. These range from sustained vasoconstriction, pulmonary vascular remodeling and *in situ* thrombosis, over heart failure, hypoxic vasoconstriction and an obliteration of the pulmonary vasculature to a prolonged occlusion of the pulmonary vascular bed (3).

As a result of the broad variety of underlying pathomechanisms and the overlapping presentation in RHC (elevated precapillary pressure in subgroups 1, 3, 4, and 5), a repertory of diagnostic tests (e.g., echocardiography, RHC, pulmonary function testing and blood gases, V/Q-scintigraphy) is recommended to achieve the final diagnosis in patients with suspected PH (1). Timely diagnosis is of crucial importance, as it not only defines therapy but also PH, regardless of the cause, is associated with poor prognosis (4–7).

With the introduction of dual energy CT (DECT), mapping of pulmonary perfusion based on the different absorption characteristics of iodine and lung parenchyma has become available (8). The generated iodine density images (IDIs) are considered a sufficient surrogate parameter to estimate organ perfusion (9, 10) and have proven to provide information on pulmonary perfusion in the setting of acute pulmonary embolism as well as in CTEPH with comparable accuracy to V/Q-scintigraphy (8, 11–18). In comparison to V/Q-scintigraphy, which remains the cornerstone to screen for CTEPH, DECT offers the advantage of allowing a comprehensive analysis of the lung parenchyma, pulmonary perfusion and vessel anatomy in a single examination (13, 16, 17, 19). With regard to the complete spectrum of PH, current data suggest that V/Q-scintigraphy (20) and DECT-based pulmonary perfusion maps might aid in the differentiation of PH subgroups, as different types of perfusion abnormalities correlate with PH etiology (21–23). In addition to the evaluation of pulmonary perfusion and vasculature in a “one-stop-examination,” DECT offers the unique possibility of quantifying parenchymal lung disease, a potential cause of PH, based on virtual non-contrast (VNC) images without the necessity of extra radiation exposure. Various studies indicate a comparable accuracy of VNC-based emphysema quantification compared to real non-contrast images (24, 25), which have also been shown to discriminate PH due to lung disease and/or hypoxia from PAH (26). As opposed to the traditional imaging approach in PH with V/Q-scintigraphy as the central modality to differentiate between CTEPH and non-CTEPH and subsequent contrast or non-contrast CT imaging for further patient characterization the integrated DECT approach yields potential to serve patient needs as well as financial considerations (27).

Notwithstanding, according to the current guidelines, DECT only plays a supportive role in the diagnostic work-up of PH (1). This appears reasonable, as its diagnostic accuracy excessively depends on reader expertise, hampering its implementation in clinical routine especially in centers with limited expertise in PH imaging (23). In addition, current data are either limited due to the small sample size and/or rely on time-consuming manual image interpretation, inheriting the limitation of intra- and interreader variability Further, hitherto most DECT studies have either assessed its ability to differentiate between health and disease or two distinct subgroups, but not the whole spectrum of PH (16, 17, 22, 23).

A recently developed software application for volumetric iodine quantification enables iodine quantification per voxel for a 3D dataset acquired on a dual-layer CT platform (spectral detector CT, SDCT) (28). This allows for a semiautomatic, threshold-based segmentation of the lung into normal and malperfused areas based on iodine concentration.

Given the potential diagnostic merit of pulmonary perfusion in PH, the aim of our study was to evaluate whether semiautomatically derived volumetric parameters of SDCT-based pulmonary perfusion maps can aid the diagnosis and classification of PH.

## Materials and Methods

### Study Population

**Figure 1:**
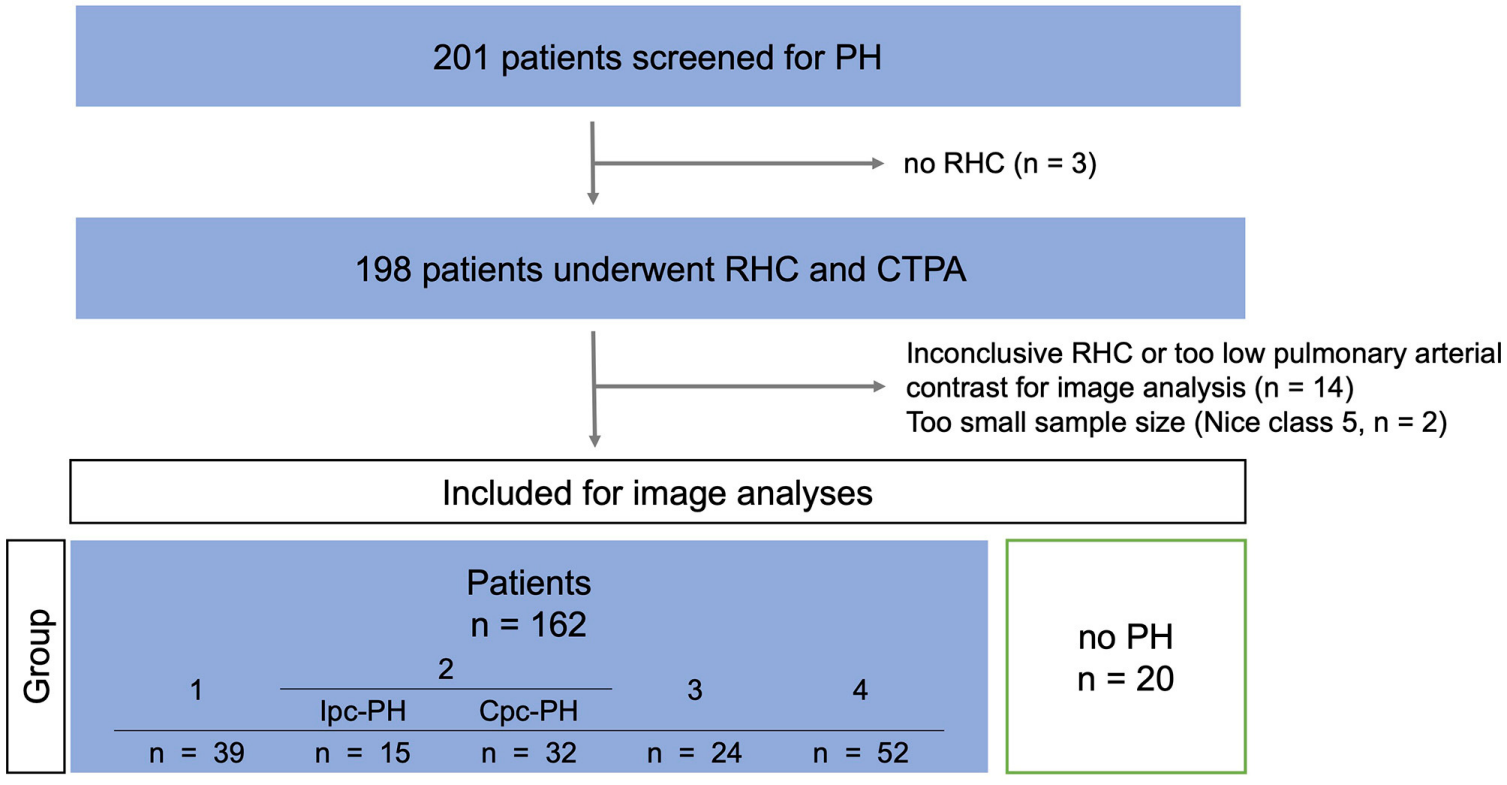
Study-flow-chart. PH, pulmonary hypertension; RHC, right heart catherization; CTPA, CT pulmonary angiography; Ipc-PH, precapillary pulmonary hypertension; Cpc-PH, combined pre- and postcapillary pulmonary hypertension.

The study cohort consisted of 201 consecutive patients who had been referred to the Department of Cardiology of the University Hospital of Cologne for PH screening between May 2016 and May 2019 and underwent CT pulmonary angiography (CTPA) on SDCT. In accordance with the 2015 ESC/ERS guidelines, patients with suspected PH underwent RHC, ventilation/perfusion scintigraphy (V/Q scan) to exclude distal CTEPH and further testing (1). CTPA and RHC were routinely performed during the same hospitalization. Patients with suspected CTEPH had to be anticoagulated for three months in advance at the time of referral. Patients with detected hypoxia had to be on stable treatment for at least four weeks before RHC and CTPA examination. The final diagnosis was reached by expert consensus based on all available diagnostic tests, including CTPA. Given the retrospective design of this study final diagnosis was based on the 2015 ESC/ERS guidelines, defining PH as an increase in the mPAP ≥ 25 mmHg (1). Twenty of these 201 patients with an invasive rule-out of PH *via* RHC (mPAP <25 mmHg) served as a control cohort (Figure 1). Among controls, besides a variety of cardiopulmonary diseases, the most frequent diagnoses were hypertensive cardiomyopathy (35.0%), chronic thromboembolic disease with no PH (15.0%) and systemic sclerosis (10.0%).

This retrospective study was approved by the local institutional review board (Ethics committee of the Faculty of Medicine from the University of Cologne, Cologne, Germany). Due to the retrospective design of the study the local institutional review board waived necessity for informed consent. All clinical investigations were conducted in accordance with the Declaration of Helsinki.

### Image Acquisition and Reconstruction

CT data were acquired on a clinically available spectral detector CT (IQon, Philips Healthcare). All patients received an intravenous 50 ml bolus of contrast media (300 mg iodine/ml, Accupaque, GE Healthcare) followed by a 40 ml NaCl flush injected with a flow rate of 4 ml/s. Scanning was initiated with a delay of 4.9 s after an attenuation of 150 HU was reached in the main pulmonary artery (MPA). The acquisition parameters were as follows: slice collimation 64 × 0.625 mm; rotation time 0.33 s; tube potential 120 kV, automatic tube current modulation was used, reference mAs 75. For all reconstructions, a soft tissue kernel (B, Philips Healthcare) and a dedicated spectral reconstruction algorithm were used (Spectral, Philips Healthcare). Images were reconstructed in axial orientation with a slice thickness of 1 mm and a slice overlap of 0.5 mm. Matrix was set to 512 × 512. In addition to conventional images, which are identical to images reconstructed with the vendors hybrid-iterative reconstruction algorithm (29) (iDose4, Philips Healthcare), iodine maps and virtual non-contrast images were reconstructed.

### Image Analysis

#### SDCT-Based Pulmonary Perfusion

**Figure 2:**
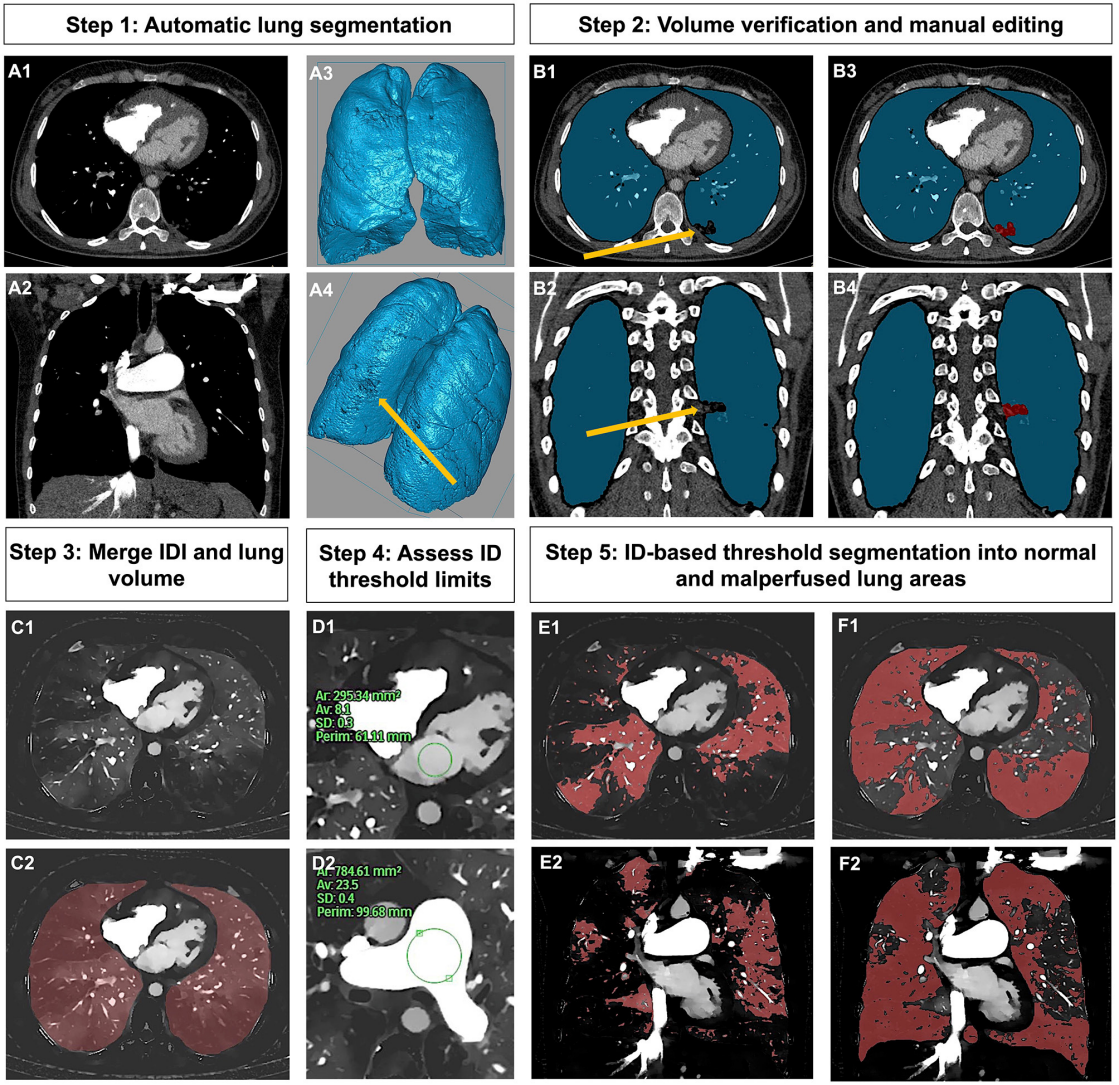
Workflow of lung segmentation into normal and malperfused lung areas. Step 1 (Panel **A1–A4**): Automatic lung segmentation. Step 2 (Panel **B1–B4**): Volume verification and in case of insufficient segmentation (yellow arrow, highlighting post-inflammatory lung alterations, which were not automatically ascribed to the lung volume) manual editing (marked in red). Step 3 (Panel **C1,C2**): Merging of IDI and lung volume. Step 4 (Panel **D1,D2**): Manual measurement of ID in the MPA and LA in order to assess segmentation thresholds. Step 5 (Panel **E1–F2**): ID-based threshold segmentation into normal (Panel **E1,E2**) and malperfused lung areas (Panel **F1,F2**). IDI, iodine density image; ID, iodine density; MPA, main pulmonary artery; LA, left atrium.

**Figure 3:**
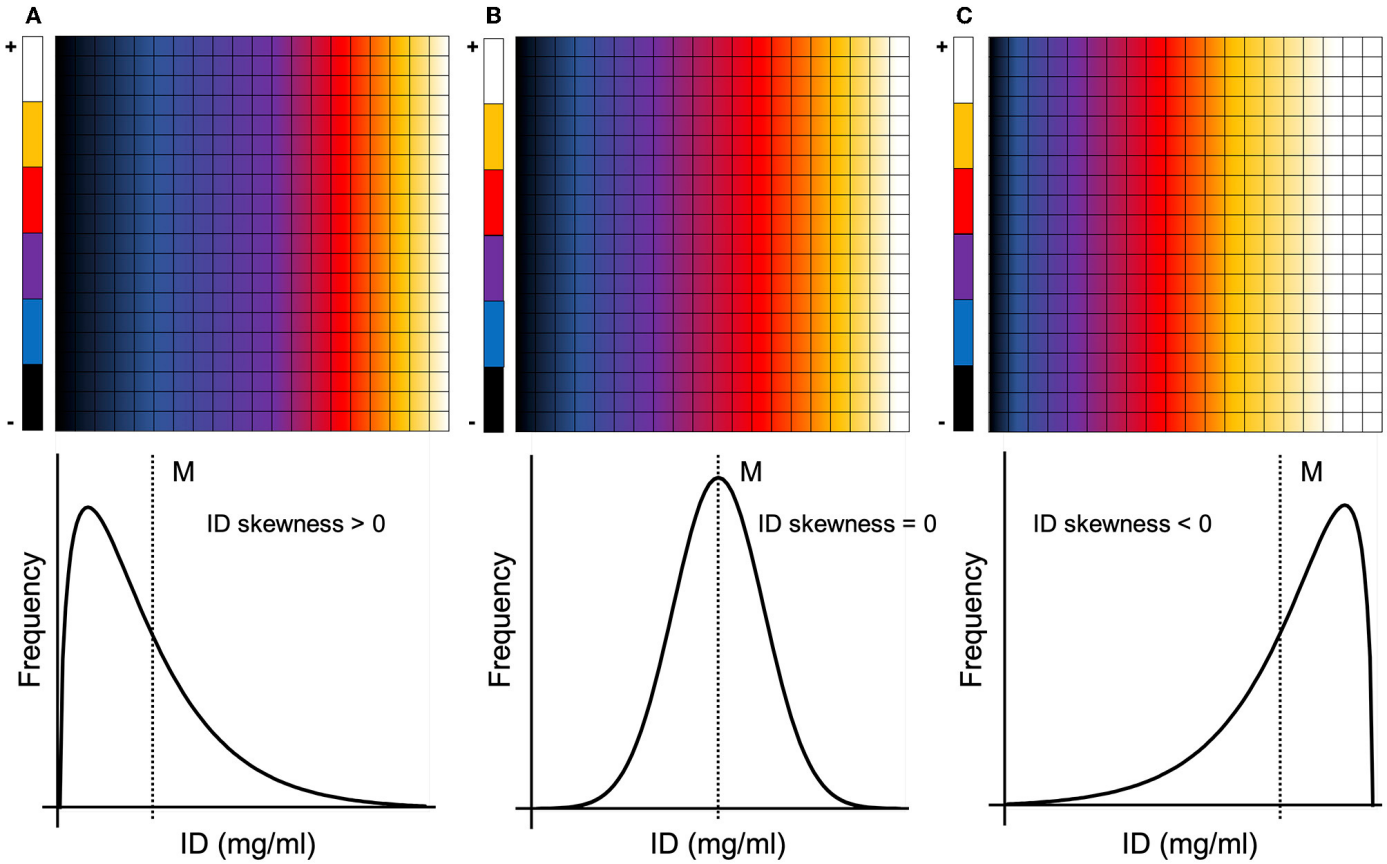
Different types of ID distributions and corresponding ID skewness. A majority of pixels/voxels with an ID below the mean result in a positively skewed ID distribution [ID skewness > 0, **(A)**], while a majority of pixels/voxels with an ID above the mean result in a negatively skewed ID distribution [ID skewness <0, **(C)**]. The more homogenous the ID distribution becomes (ultimately tending toward a normal distribution), the more the ID skewness approaches zero [ID skewness = 0, **(B)**]. M, mean; ID, iodine density.

Automatic segmentation of the lung was performed using the commercially available software solution (Intellispace Portal, COPD tool, Version 10, Philips Healthcare). All segmentations were manually verified by a radiologist and in case of insufficient segmentation, lung volumes were manually edited. Subsequently, lung volumes were semiautomatically segmented into normal and malperfused areas *via* threshold segmentation based on the IDIs using dedicated software for volumetric iodine quantification (ISD, ThresholdSegmentation (1.1) Philips Intellispace Release 11). Three lung areas were defined as previously described (30), corresponding thresholds given brackets: malperfused (PerfDef): voxels with an iodine density (ID) of ≤ 5 % of the MPA (PerfDef = 0.0– ≤ 5 % ID of the MPA), normal perfused (PerfNorm): voxels with an ID of more than 5% of the MPA and ≤ 50 % of the left atrium (LA) (PerfNorm ≥5 % MPA— ≤ 50 % LA), vessel compartment (Ves): voxels with an ID of more than 50% of the LA (Ves = > 50 % MPA−100). Mean IDs in the MPA and LA were measured by manually drawing a ROI that accounted for at least 50% of the vascular/atrial area at the largest diameter. The following parameters were computed from histogram analysis for normal and malperfused lung areas: mean ID, ID kurtosis and ID skewness. Values for mean ID are given as standardized values calculated by division by the mean ID of the feeding vessel (MPA), as described previously (31). Figure 2 visualizes the segmentation workflow. Figure 3 illustrates the effect of ID distribution on ID skewness.

#### Lung Emphysema Quantification

Lung emphysema was quantified based on VNC images using a commercially available software solution (Intellispace Portal, COPD tool, Version 10, Philips Healthcare). The tool makes use of a threshold-based emphysema calculation approach applying a threshold of −950 Hounsfield units (HU). Insufficiently segmented lung volumes were manually reedited (Supplementary Figure 1).

#### ID Skewness_PerfDef_-Emphysema-Index

The ID skewness in malperfused lung areas (ID Skewness_PerfDef_)-Emphysema-Index (δ-index) reflecting the ratio of ID distribution in malperfused lung areas and the proportion of emphysematous lung parenchyma was calculated forming the quotient of these two parameters. Discrete translation was performed to the parameter ID Skewness_PerfDef_ by more than the smallest negative measured value (−1.35) to ensure that the data distribution was transformed in a strictly positive range. To obtain a calculable devisor, the proportion of emphysematous lung parenchyma was also transformed.


$$\begin{array}{l} \;{\text{ID Skewness}}_{\text{PerfDef}}-\text{Emphysema}-\text{Index }\left(\delta -\text{Index}\right)\\ =\frac{\text{ID Skewnes}{\text{s}}_{\text{PerfDef}}+1.36}{\text{Proportion of emphysematous lung parenchyma}+1.0} \end{array}$$

The δ-index aims at distinguishing between homogenous pulmonary perfusion defects caused by parenchymal lung alterations and perfusion defects caused by true vascular malperfusion, e.g., due to vascular narrowing and/or obstruction as seen in CTEPH. While in both cases the homogenous ID distribution within the perfusion defect leads to an ID skewness close to zero (Figure 3), the index will either in- or decrease, depending on the amount of emphysematous lung alterations.

Table 1 gives an overview over all parameters as assessed from RHC and SDCT.

**Table 1:** Assessed RHC and SDCT parameters.

**RHC**	**SDCT**
RA pressure [mmHg]	Pulmonary perfusion*
SvO2 [%]	Volume [% of total lung volume]
CI [l/min/m^2^]	Mean ID
mPAP [mmHg]	ID Kurtosis
PVR* [mmHg]	ID Skewness
PCWP [mmHg]	
	**Pulmonary parenchyma**
	Emphysema [% of total lung volume]
	δ-index

RHC, right heart catherization; SDCT, spectral detector CT; RA, right atrial; SvO_2_, pulmonary arterial oxygen saturation (SvO^2^); CI, cardiac index; mPAP, mean pulmonary artery pressure; PVR, pulmonary vascular resistance; PCWP, pulmonary capillary wedge pressure; ID, iodine density; ^*^in normal and malperfused lung areas.

### Statistical Analysis

Normal distribution was tested using the Shapiro-Wilk test. Differences in continuous, parametric data were compared using the *t*-test. Continuous, independent, non-parametric data were compared using the Mann-Whitney *U* test. Differences in categorical data were identified using Pearson's chi-squared test. Variances among and between the subgroups concerning continuous data were compared using ANOVA for parametric data. After assessing the equality of variances using Leven's test, *post hoc* testing was performed by Bonferroni adjustment for multiple comparisons. On the other hand, differences between the subgroups for categorial, non-parametric variables were assessed using the Kruskal-Wallis test and Dunn-Bonferroni corrected *post hoc* analysis. The area under the receiver operating characteristic curve (AUC) was calculated for subclassification. Based on AUC analysis parameters, sensitivity and specificity for subclassification were calculated using Youden's index.

A *p*-value of <0.05 was considered statistically significant. Statistical analysis was performed using SPSS software (IBM SPSS Statistics for macOS, Version 27.0, Armonk, NY, USA).

## Results

### Study Population

All patient demographics are given in Table 2 and Supplementary Table 1. 201 patients were screened for PH. Of these, three patients were not eligible for RHC, and 14 had an inconclusive RHC or too low pulmonary arterial contrast for image analyses, defined by an ID in the MPA <5 mg/mL. Subgroup 5 was excluded because of its small sample size (*n* = 2). In 20 patients, RHC ruled out PH (mPAP <25 mmHg), and these patients served as controls (Figure 1).

**Table 2:** Baseline characteristics.

	**PH**	**Controls**	***p***
**Parameter**	**(*n* = 162)**	**(*n* = 20)**	
Age [years]	70 (57–77)	64 (57–78)	0.82
Sex [m/f]	60/102	7/13	0.12
NYHA class			0.32
1	1	0	
2	17	3	
3	102	14	
4	13	0	
NT-pro BNP	918 (303–2160)	338 (154–620)	**<0.01**
**RHC**			
RA pressure [mmHg]	8.0 (5.0–11.0)	4.0 (2.0–7.0)	**0.001**
SvO2 [%]	64.3 (59.8–68.2)	67.8 (63.1–71.0)	**0.04**
CI [l/min/m^2^]	2.2 (1.9–2.6)	2.4 (2.1–3.0)	**0.049**
mPAP [mmHg]	40.5 (32.8–50.0)	21.0 (18.3–23.0)	**<0.001**
PVR* [mmHg]	6.1 (3.9–9.6)	2.1 (1.5–2.6)	**<0.001**
PCWP [mmHg]	14.0 (10.0–18.3)	10.5 (9.0–12.0)	0.01
**Echocardiography**			
RA area [cm^2^]	22.0 (19.0–28.6)	22.0 (17.0–27.9)	0.42
TAPSE [mm]	20.0 (16.1–23.0)	22.0 (18.0–24.0)	0.25
TR velocity [m/s]	4.0 (3.4–4.5)	3.1 (2.8–3.3)	**0.02**
RVEDD [mm]	42.0 (38.0–47.9)	38.8 (32.5–46.3)	**0.03**
sPAP [mmHg]	70.4 (54.5–87.3)	43.0 (39.8–58.4)	**<** **0.01**

Data are given as the mean (IQR). PH, pulmonary hypertension; NYHA class, New York Heart Association (NYHA) Functional Classification (32); NT-pro BNP, N-terminal pro-B-type natriuretic peptide; RHC, right heart catherization; RA, right atrial; SvO_2_, pulmonary arterial oxygen saturation (SvO^2^); CI, cardiac index; mPAP, mean pulmonary artery pressure; PVR, pulmonary vascular resistance; PCWP, pulmonary capillary wedge pressure; TAPSE, tricuspid annular plane systolic excursion; TR, tricuspid regurgitation; RVEDD, right ventricular end-diastolic diameter; sPAP, systolic pulmonary artery pressure; ^*^defined as: (mPAP—PCWP)/cardiac output. The bold values indicate the significant differences.

### SDCT-Based Differentiation Between PH Patients and Controls

Table 3 displays all SDCT-based lung perfusion parameters in normal and malperfused lung areas for patients with PH and controls. Based on the ID in the MPA and the LA, right-to-left-heart contrast transit *via* the pulmonary vasculature was similar between groups. Patients suffering from PH showed higher proportions of malperfused lung areas (*p* = 0.02) as well as stronger (mean ID, *p* = 0.03) and more homogenous perfusion deficits (ID skewness, *p* = 0.03). Neither ID skewness in normal perfused lung areas nor ID kurtosis in any lung compartment differed significantly between groups. Based on AUC analysis the proportion of normal perfused lung areas qualified as the best SDCT-parameter to identify patients with PH (AUC: 0.67, 95%-CI, 0.56–0.78, sensitivity, 74%, specificity 55%, applying a cut-off of 71,6% as determined by Youden's index), yielding comparable diagnostic accuracy to the NT-pro BNP (AUC: 0.69, 95%-CI, 0.57–0.80, sensitivity, 59%, specificity 80%). At balanced sensitivities for both parameters the proportion of normal perfused lung areas was more specific than the NT-pro BNP (55 vs. 50%) (Table 4).

**Table 3:** SDCT-based pulmonary perfusion in patients and controls.

	**PH**	**Controls**	***p***
**Parameter**	**(*n* = 162)**	**(*n* = 20)**	
ID MPA	14.1 (11.6–16.9)	15.1 (11.7–17.1)	0.93
ID LA	8.9 (7.2–10.5)	9.6 (8.4–12.4)	0.07
**Normal perfused lung**			
Volume [% of total lung volume]	57.1 (37.5–73.6)	72.6 (55.9–79.9)	**0.01**
Mean ID	0.091 (0.083–0.109)	0.095 (0.085–0.121)	0.52
ID Kurtosis	4.67 (2.45–7.42)	6.77 (2.63–9.72)	0.16
ID Skewness	1.98 (1.63–2.50)	2.43 (1.42–2.84)	0.27
**Malperfused lung**			
Volume [% of total lung volume]	39.0 (24.0–59.7)	25.6 (16.8–42.7)	**0.02**
Mean ID	0.026 (0.021–0.030)	0.028 (0.025–0.032)	**0.03**
ID Kurtosis	−1.00 (−1.25 to −0.62)	−1.11 (−1.33 to −0.16)	0.77
ID Skewness	−0.30 (−0.62–0.08)	−0.47 (−0.85 to −0.26)	**0.03**

*Data are given as the mean (IQR). SDCT, spectral detector CT; PH, pulmonary hypertension; ID, iodine density; MPA, main pulmonary artery; LA, left atrium. The bold values indicate the significant differences*.

**Table 4:** Diagnostic accuracy of SDCT-based pulmonary perfusion parameters and NT-pro BNP.

**Parameter**	**AUC**	**95%-CI**	**Sensitivity**	**Specificity**
NT-pro BNP	0.69	0.57–0.80	59	80
			74*	50*
**Pulmonary Perfusion**				
**Normal perfused lung**				
Volume [% of total lung volume]	0.67	0.56–0.78	74	55
**Malperfused lung**				
Volume [% of total lung volume]	0.66	0.55–0.78	67	60
Mean ID	0.65	0.53–0.76	58	70
ID Skewness	0.65	0.53–0.77	53	75

SDCT, spectral detector CT; AUC, area under the receiver operating characteristic curve; CI, confidence interval; ID, iodine density. ^*^at a set sensitivity equal to normal perfused lung volume.

### SDCT-Based Differentiation of PH Subgroups

The Kruskal-Wallis test revealed significant differences between PH subgroups for the percentage of normal perfused lung volume (V_PerfNorm_) and malperfused lung volume (V_PerfDef_), mean ID, ID kurtosis as well as ID skewness in malperfused lung areas (*p* for all <0.001) (Table 5). The comparison for all baseline characteristics between PH subgroups is given in Supplementary Table 2.

**Table 5:** SDCT-based pulmonary perfusion and pulmonary parenchyma characteristics of different PH subgroups.

	**Group**					
**Parameter**	**1**	**2**		**3**	**4**	***p***
		**Ipc-PH**	**Cpc-PH**			
	**(*n* = 39)**	**(*n* = 15)**	**(*n* = 32)**	**(*n* = 24)**	**(*n* = 52)**	
**Pulmonary perfusion**						
**Normal perfused lung**						
Volume [% of total lung volume]	62.9 (41.1–78.2)	75.9 (64.8–87.7)	57.6 (39.5–70.4)	42.2 (20.9–58.9)	55.4 (39.2–70.4)	<0.001*
Mean ID	0.090 (0.081–0.104)	0.115 (0.080–0.135)	0.089 (0.083–0.100)	0.092 (0.085–0.112)	0.093 (0.085–0.111)	0.48
ID Kurtosis	5.03 (2.43–6.77)	4.51 (3.45–9.49)	5.22 (2.35–8.19)	3.64 (2.34–7.14)	4.56 (3.01–7.40)	0.84
ID Skewness	2.15 (1.63–2.64)	1.84 (1.67–2.68)	2.10 (1.62–2.66)	1.86 (1.59–2.48)	1.90 (1.57–2.44)	0.88
**Malperfused lung**						
Volume [% of total lung volume]	33.7 (18.3–56.6)	21.4 (7.7–31.1)	37.2 (26.7–58.8)	53.7 (37.2–77.9)	42.5 (27.8–59.1)	**<0.001**†
Mean ID	0.029 (0.026–0.033)	0.029 (0.025–0.032)	0.028 (0.024–0.031)	0.021 (0.017–0.025)	0.023 (0.020–0.027)	**<0.001**
ID Kurtosis	−0.77 (−0.98 to −0.16)	−0.98 (−1.28 to −0.05)	−0.85 (−1.21 to −0.35)	−1.21 (−1.39 to −0.93)	−1.14 (−1.26 to −0.97)	**<0.001**‡
ID Skewness	−0.58 (−0.87 to −0.13)	−0.57 (−0.85 to −0.22)	−0.47 (−0.74 to −0.19)	0.03 (−0.23–0.54)	−0.05 (−0.33–0.18)	**<** **0.001**
**Pulmonary parenchyma**						
Emphysema [% of total lung volume]	0.2 (0.0–1.8)	0.1 (0.1–0.2)	0.3 (0.1–2.0)	8.4 (2.8–17.1)	0.4 (0.1–1.3)	**<0.001**§
δ-index	0.43 (0.27–0.66)	0.72 (0.42–1.03)	0.44 (0.20–0.62)	0.17 (0.10–0.34)	0.75 (0.55–1.10)	**<0.001**||
Co-existing lung disease [n/n]	14/39 (35.9%)	6/15 (40.0%)	11/32 (34.4%)	24/24 (100.0%)	13/52 (25.0%)	**<0.001**|||

Data are given as the mean (IQR). SDCT, spectral detector CT; Ipc-PH, isolated precapillary pulmonary hypertension; Cpc-PH, combined pre- and postcapillary pulmonary hypertension; ID, iodine density, δ-index, ID Skewness_PerfDef_-Emphysema-Index. ^*^ 3-Ipc-PH, p <0.001; 4-Ipc-PH, p = 0.02, † 3-Ipc-PH, p <0.001; 4-Ipc-PH, p = 0.01, ‡ 3–1, p <0,01; 4–1, p <0.01, § 3–1; 3-Ipc-PH; 3-Cpc-PH; 3–4, p for all <0.001, || 3-Ipc-PH, p <0.001, 3–4, p <0.001; 4–1, p <0.01; 4-Cpc-PH, p <0.01, ||| 3–1; 3-Ipc-PH; 3-Cpc-PH; 3-4, p for all <0.001. The bold values indicate the significant differences.

**Figure 4:**
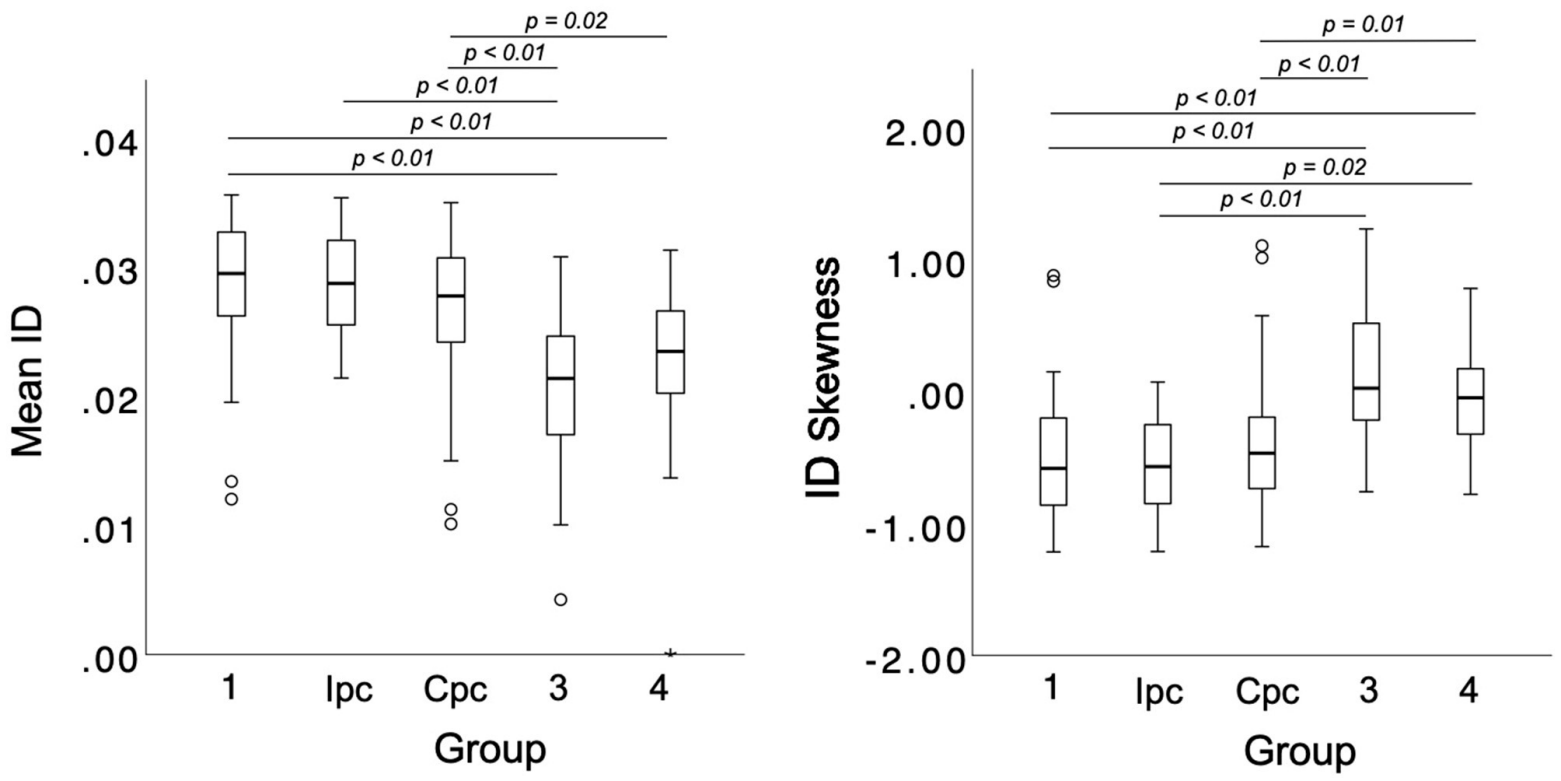
SDCT-based differentiation of PH subgroups according to mean ID and ID skewness in malperfused lung areas. ID, iodine density; Ipc, precapillary pulmonary hypertension; Cpc, combined pre- and postcapillary pulmonary hypertension.

Patients suffering from PH due to chronic lung disease and/or hypoxia had the largest extent of V_PerfDef_ among all subgroups, with a significantly higher proportion of malperfused lung areas compared to patients with Ipc-PH (Table 5). Moreover, malperfused lung areas showed a significantly stronger (mean ID) and more homogenous perfusion deficit (ID skewness) compared to all other subgroups (*p* for all <0.001, Figure 4), except for subgroup 4 (*p* = 1.00, respectively).

**Figure 5:**
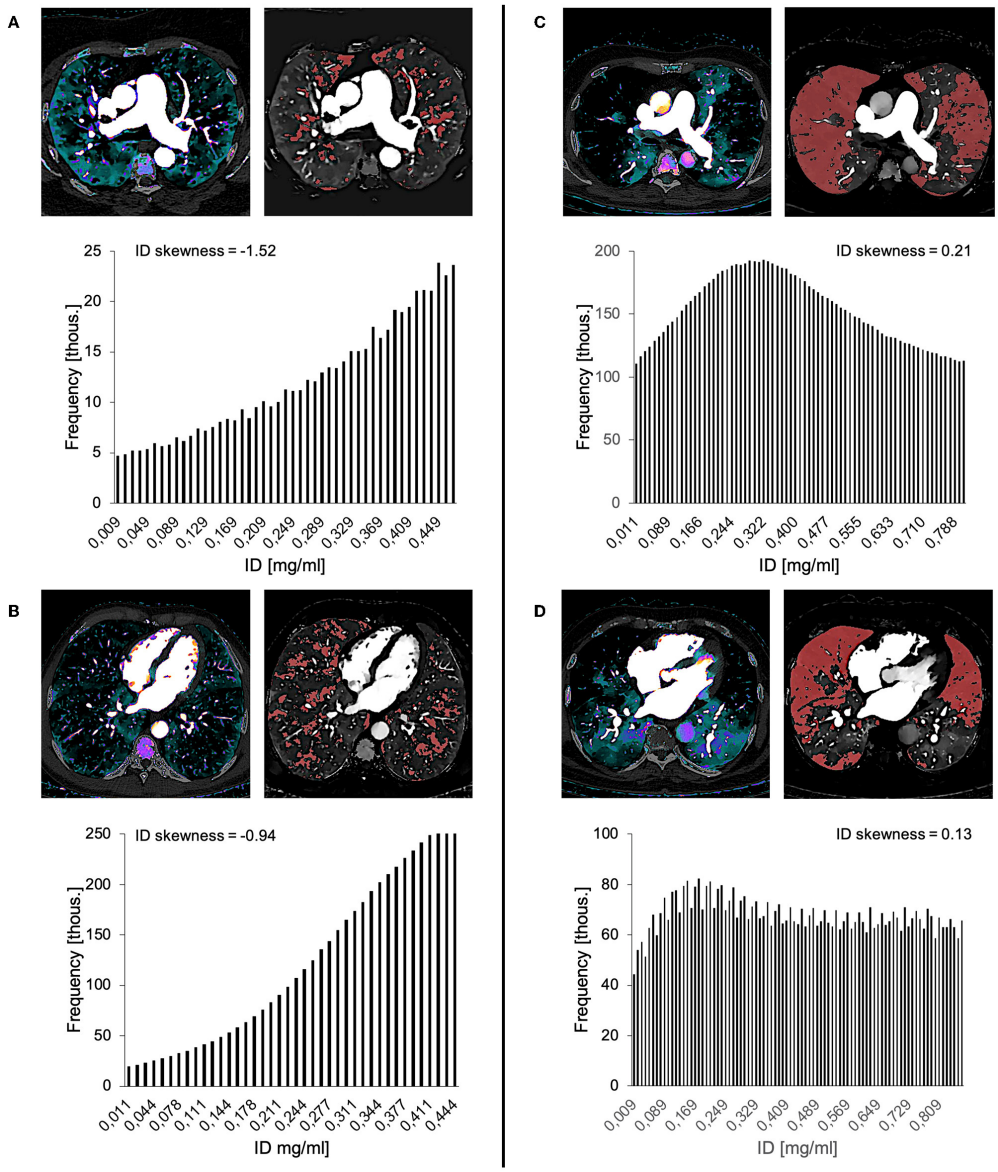
IDIs with automatically derived malperfused lung areas (red) and corresponding ID histograms of the perfusion deficit volume illustrating the difference of pattern and ID distribution of malperfused lung areas in two example cases of PAH **(A,B)** and CTEPH **(C,D)**, respectively. Corresponding histograms indicate an inhomogeneous ID distribution in PAH (ID skewness <0) and a more homogenous ID distribution in CTEPH (ID skewness close to 0). Thous., thousand; ID, iodine density.

Corresponding results were demonstrated for patients with CTEPH showing a significantly stronger perfusion deficit as well as more homogenous ID distribution in malperfused lung areas compared to all other subgroups (*p* for all <0.001, only for mean ID 4–6 *p* = 0.01, Figure 4), except subgroup 3 (*p* = 1.00). ID skewness in malperfused lung areas allowed for an accurate identification of subgroups 3 and 4 based on AUC analysis (AUC: 0.79, 95%-CI, 0.72–0.86). Applying a cut-off of −0.335 as determined by Youden's index, a sensitivity of 84% and a specificity of 70% could be achieved for subclassification. Figure 5 illustrates the difference in pattern and ID distribution of malperfused lung areas in patients with PAH and patients suffering from CTEPH.

Neither normal nor malperfused lung areas revealed any difference between group 1 and patients suffering from Ipc-PH or Cpc-PH.

**Figure 6:**
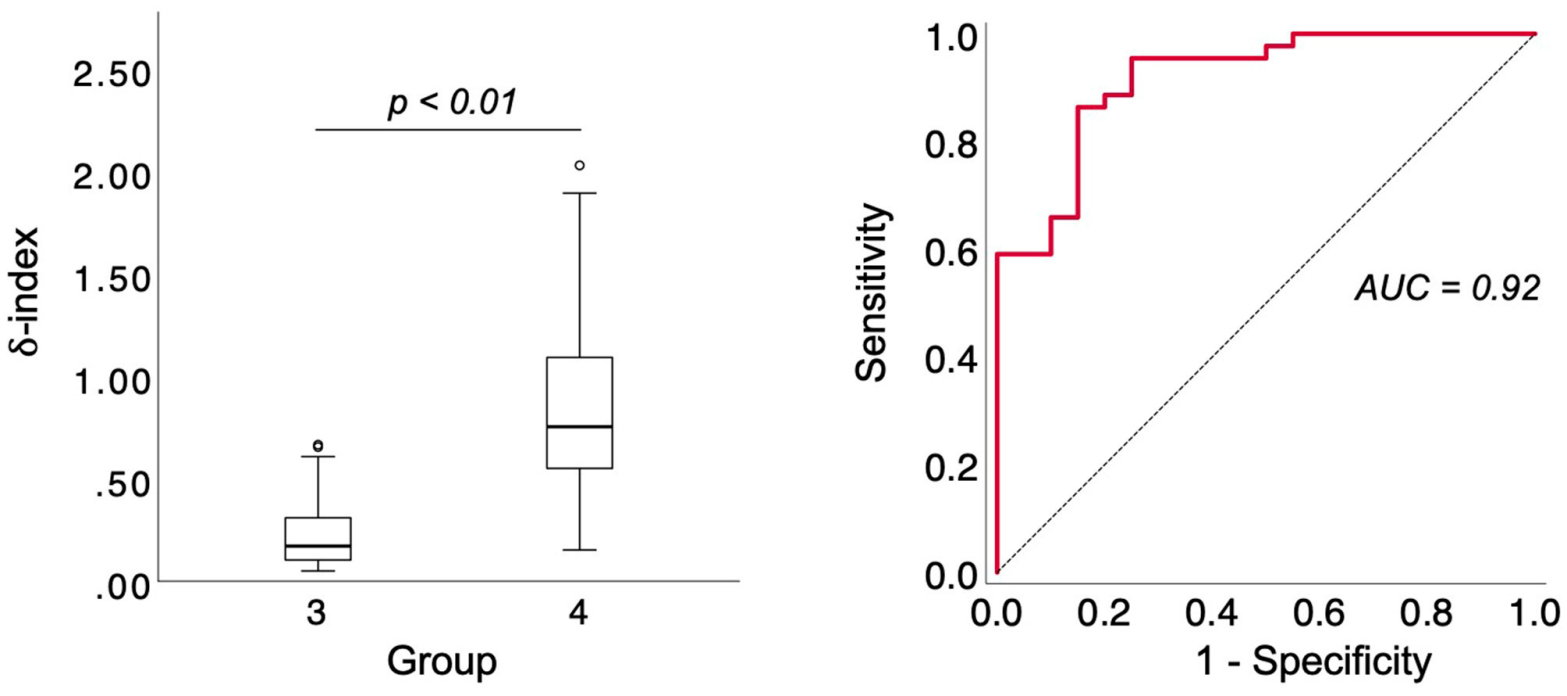
Differentiation of patients with PH due to lung disease and/or hypoxia and patients with CTEPH based on the δ-index and diagnostic accuracy of the δ-index for CTEPH based on AUC analysis. CTEPH, chronic thromboembolic pulmonary hypertension; δ-index, ID Skewness_PerfDef_-Emphysema-Index.

**Figure 7:**
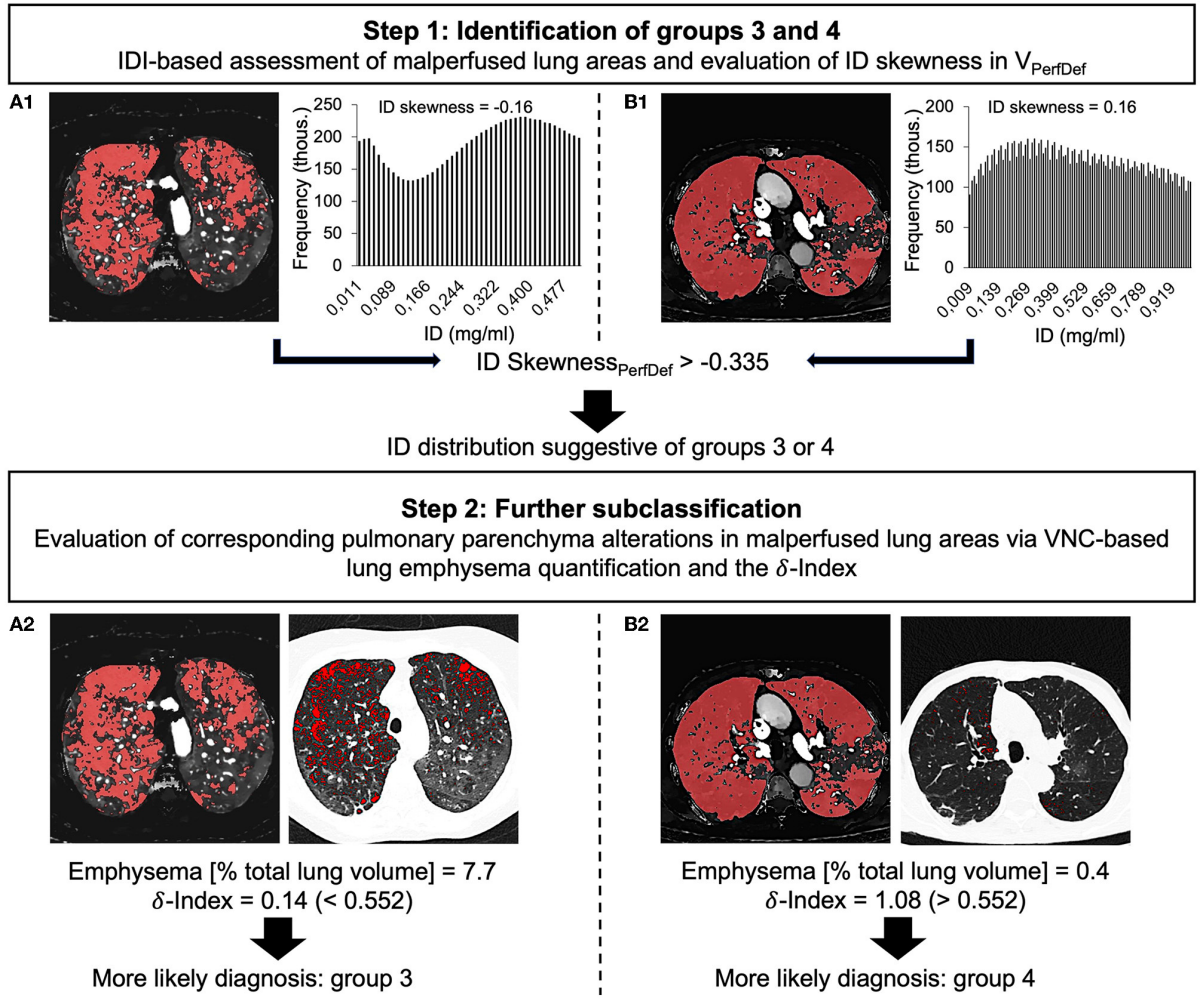
Stepwise approach for the identification and differentiation of groups 3 **(A1,A2)** and 4 **(B1,B2)** based on ID skewness in malperfused lung areas, VNC-based quantification of emphysematous lung parenchyma and the δ-index. IDI, iodine-density image; ID, iodine density; V_PerfDef_, malperfused lung volume; thous., thousand; (ID Skewsness_PerfDef_, ID skewness in malperfused lung areas; VNC, virtual non-contrast, δ-index, ID Skewness_PerfDef_-Emphysema-Index.

Excluding group 3 the prevalence of co-existing lung disease varied between 25.0% and 40.0% among subgroups (Table 5). 8 patients (33.3%) from subgroup 3 were suffering from parenchymal lung disease other than chronic obstructive pulmonary disease (COPD). Patients from subgroup 3 with COPD did not show a higher amount of emphysematous lung alterations as assessed based on VNC reconstructions than those with lung fibrosis (*p* = 0.28). The δ-index differed significantly between subgroups 3 and 4 (Table 5; Figure 6). Based on AUC analysis, the index allowed for an accurate identification of patients with CTEPH (AUC: 0.92, 95%-CI, 0.85–0.99). A cut-off of 0.502 as determined by Youden's index was best for subclassification (sensitivity 86%, specificity 85%). The diagnostic accuracy of the δ-index was not affected by the presence of lung fibrosis (AUC: 0.92, 95%-CI, 0.84–1.00). The stepwise differentiation of both subgroups based on IDIs and VNC-based lung emphysema quantification is exemplified in Figure 7.

## Discussion

With the to our knowledge, the first study evaluating the potential of SDCT-derived pulmonary perfusion maps and pulmonary parenchyma characteristics for the semiautomated diagnosis and classification of PH with differing etiologies we can present several notable findings. (1) Semiautomatic lung segmentation into normal and malperfused lung areas based on ID values is feasible and applicable in the setting of PH. (2) SDCT-based pulmonary parameters can not only detect but also quantify pulmonary perfusion abnormalities in PH holding comparable accuracy for PH diagnosis as NT-pro BNP. (3) ID skewness in malperfused lung areas and the introduced δ-index could potentially yield merit for a semiautomatic identification of PH subgroups, especially for the subclassification of groups 3 and 4.

### Differentiation of PH Patients and Controls

The accurate diagnosis of PH remains challenging (33) and no meaningful decrease in the time from symptom onset to diagnosis could be achieved throughout the last decades (26). This also accounts for the high morbidity and mortality of PH, regardless of the underlying etiology (4–7). Although RHC is accompanied by peri- and postinterventional complications and does not allow for morphologic information (34), it remains the diagnostic gold standard (1), as non-invasive imaging parameters either lack diagnostic accuracy (33, 35, 36) or have not yet been validated in large study cohorts, albeit promising initial results (37, 38). This is in line with our findings. Although our data demonstrate that SDCT can not only assess but also quantify pulmonary malperfusion in PH, the diagnostic implications of these findings seem to be limited: while the volume of normal perfused lung areas yielded comparable diagnostic accuracy to the NT-pro BNP and thus might aid the detection of disease, the overall diagnostic accuracy of the SDCT-based pulmonary perfusion parameters was only moderate. Moreover, subgroup analysis revealed no significant difference in any volumetric pulmonary perfusion parameter between group 1 and Ipc-PH and controls (*p* for all > 0.05, data not shown). Concurrently, Kim et al. found only 60.0%, and Giordano et al. even found only 52.6% of PAH patients showing abnormal pulmonary perfusion (21, 22). Although it remains unclear to what extent our results might be affected by the composition of the control group, which consisted of patients with the clinical suspicion of PH, a variety of cardio-pulmonary diseases and a borderline mPAP of 21.0 mmHg, our data thus underscore the challenges of a non-invasive PH diagnosis.

### Classification of PH Subgroups

Our results demonstrate the merit of SDCT-derived pulmonary perfusion maps and pulmonary parenchyma characteristics over and above RHC for the classification of PH subgroups, particularly the spectrum of precapillary PH, which remains the main task from the radiologist's view (22).

There is evidence from V/Q-scintigraphic and DECT-based studies that pulmonary perfusion maps might enable the differentiation of PH subgroups, as different types of perfusion abnormalities correlate with PH etiology (20–23). Regardless of the modality, these studies are limited due to small sample sizes and/or time-consuming quantitative or semiquantitative image interpretation with considerable intra- and interreader variability. We demonstrated that semiautomatically generated volumetric parameters of SDCT-based pulmonary perfusion maps can aid the classification of PH. On the basis of ID skewness in malperfused lung areas, patients suffering from PH due to chronic lung disease and/or hypoxia and patients with CTEPH could confidently be differentiated from all other subgroups. Corresponding to the characteristic multisegmental, sharply defined, wedge-shaped and hypoattenuated appearance (17, 39), which is also described for PE (12) and is pathophysiologically caused by either acute (PE) or prolonged (CTEPH) occlusion of the pulmonary arteries (3, 40), malperfused lung areas in patients with CTEPH stood out due to more pronounced and more homogenous perfusion defects. The same was true for patients with PH due to chronic lung disease and/or hypoxia, which can be explained by the emphysematous lung changes prevalent in this patient group and the consecutive hypoxic vasoconstriction and obliteration of the pulmonary vascular bed (3) leading to large areas with no iodine attenuation at all. On the contrary pulmonary perfusion defects in groups 1 and 2 revealed a more heterogenous ID distribution as compared to groups 3 and 4, reflecting the more diffuse and less regional nature of pulmonary vascular remodeling in these groups.

Together with subgroups 1 and 5, subgroups 3 and 4 form the spectrum of precapillary PH. The subclassification of precapillary PH marks a key point in the diagnostic work-up of PH, as the distinct forms cannot be differentiated *via* RHC (1). In this context, the identification of patients with CTEPH plays a pivotal role since these patients face a particularly poor prognosis (41) and can potentially be treated by surgical thrombendarteriectomy or balloon angioplasty. The evaluation of DECT-derived pulmonary perfusion maps for the diagnosis of CTEPH has been validated in numerous studies against V/Q-scintigraphy (16, 17), which is considered the gold standard to screen for the disease (1). With the introduction of the SDCT-based δ-index, we were able to identify a powerful parameter that allows for the semiautomated identification of patients with CTEPH. Contrary to previous SDCT studies differentiating CTEPH from other forms of PH (16, 17, 23), this approach works independently of reader expertise. This might be helpful to facilitate confident diagnosis of CTEPH for radiologists with limited expertise in PH imaging. In comparison to V/Q-scintigraphy, it overcomes the modality's inherent main limitation of V/Q-scintigraphy, which is unable to obtain morphologic information such as parenchymal changes or vessel anatomy (13, 16, 17, 19). In combination with the interpretation of the pulmonary vasculature, SDCT-based pulmonary perfusion maps might thus aid the identification of patients with CTEPH and a high probability of profiting from treatment (22). Unlike a sequential approach of V/Q-scintigraphy and conventional CTA, the integrated SDCT approach offers the unique possibility of quantifying parenchymal lung disease based on VNC images without the necessity of extra radiation exposure (24, 42). The δ-index thus automatically accounts for the radiologist's task to interpret iodine maps in correlation with pulmonary parenchyma alterations to differentiate between true perfusion defects and perfusion defects due to pulmonary pathologies such as emphysema (41).

### Limitations

Apart from the retrospective study design and the missing validation of our findings, several limitations need to be addressed. First, the strict indication for an RHC intervention led to a considerable selection bias of the control group, which did not consist of truly healthy individuals but patients with a clinical suspicion of PH, a variety of cardio-pulmonary diseases and a borderline mPAP. Noteworthy, only 8 of 20 controls had a mPAP ≤ 20 mmHg. On the other hand, the invasive characterization of the controls is a strength of this study. Notwithstanding, a validation of our findings in controls with an mPAP <20 mmHg is highly desirable, especially in the light of the upcoming change in the hemodynamic definition of PH (2). Second, the inability of the δ-index to differentiate between true perfusion defects and pseudodefects, e.g., due to beam hardening or motion artifacts, is a considerable limitation (43). Third, the δ-index does not allow for a direct quantification of other parenchymal lung disease than lung emphysema, e.g., lung fibrosis. Its diagnostic accuracy thus indirectly depends on the co-existence of emphysematous and non-emphysematous parenchyma alterations. Worth mentioning, in group 3 there was no significant difference between patients with and patients without lung fibrosis regarding the amount of emphysematous lung alterations, which is in line with the increasingly recognized coexistence of both diseases (44, 45). Further, the presence of lung fibrosis did not have a negative influence on the diagnostic accuracy of the δ-index for the differentiation between groups 3 and 4. Fourth, due to scan timing, SDCT-derived perfusion maps only provide very limited information on lung compartments with maintained blood supply beyond the occluded pulmonary arteries through systemic collateral blood flow, e.g., *via* bronchial arteries, best described for CTEPH (41), and thus might overestimate the degree of perfusion deficit. This probably represents the methods biggest limitation. Fifth, we did not differentiate between major-vessel and minor-vessel CTEPH, which is known to partly mimic typical PAH perfusion deficit patterns (22). Validation of our findings in a PAH and subclassified CTEPH population thus seems highly desirable. Last, our data did not allow for an independent validation of the VNC-based emphysema quantification, as the CTPA protocol did not include true non-contrast images. Further studies addressing this considerable limitation are highly warranted.

### Conclusion

SDCT-derived pulmonary perfusion and pulmonary parenchyma characteristics can detect and quantify pulmonary perfusion abnormalities in PH and allow for a semiautomated diagnosis of groups 3 and 4, independent of reader expertise.

## Data Availability Statement

The raw data supporting the conclusions of this article will be made available by the authors, without undue reservation.

## Ethics Statement

The studies involving human participants were reviewed and approved by Ethics Committee of the Faculty of Medicine from the University of Cologne, Cologne, Germany. Written informed consent for participation was not required for this study in accordance with the national legislation and the institutional requirements.

## Author Contributions

RG performed the data collection, image analysis, statistical analysis, and the writing of the manuscript including graphic preparation. JKr and RG supported image analysis. FG and SR carried out invasive testing. RS and LC performed image feature extraction and analysis. JKr and AB supported study design. JKo and RG supported statistical analysis. NH, AB, and DM supported graphic preparation and manuscript conceptualization. AB carried out project supervision. All authors reviewed the manuscript. All authors contributed to the article and approved the submitted version.

## Conflict of Interest

RG received research support from Philips Healthcare. NH has been supported by the Else Kröner-Fresenius-Stiftung (2018_EKMS.34 to NH) and receives research support from Philips Healthcare. NH and DM are on the speaker's bureau of Philips Healthcare. RS is employed by Philips Healthcare. The remaining authors declare that the research was conducted in the absence of any commercial or financial relationships that could be construed as a potential conflict of interest. The reviewer AR declared a past co-authorship with one of the authors SR to the handling Editor.

## Publisher's Note

All claims expressed in this article are solely those of the authors and do not necessarily represent those of their affiliated organizations, or those of the publisher, the editors and the reviewers. Any product that may be evaluated in this article, or claim that may be made by its manufacturer, is not guaranteed or endorsed by the publisher.

## Abbreviations

AUC: Area under the receiver operating characteristic curve
CI: Confidence interval
CO: Cardiac output
COPD: Chronic obstructive pulmonary disease
Cpc-PH: Combined pre- and postcapillary pulmonary hypertension
CTEPH: Chronic thromboembolic pulmonary hypertension
CTPA: CT pulmonary angiography
DECT: Dual energy CT
DPG: Diastolic pressure difference
ID: Iodine density
IDI: Iodine density image
Ipc-PH: Isolated postcapillary pulmonary hypertension
LA: Left atrium
MPA: Main pulmonary artery
mPAP: Mean pulmonary artery pressure
PAH: Pulmonary arterial hypertension
PCWP: Pulmonary capillary wedge pressure
PerfDef: Perfusiondeficit/malperfused lung areas
PerfNorm: Normalperfused lung areas
PH: Pulmonary hypertension
PVR: Pulmonary vascular resistance
RA: Right atrial
RHC: Right heart catheterization
RVEDD: Right ventricular end-diastolic diameter
SDCT: Spectral detector CT
sPAP: Systolic pulmonary artery pressure
SvO^2^: Pulmonary arterial oxygen saturation
TR velocity: Tricuspid regurgitation velocity
TTE: Transthoracic echocardiography
VNC: Virtual non-contrast
δ-index: ID Skewness_PerfDef_-Emphysema-Index.
