# Supplementary material for: Spectral Detector CT-Derived Pulmonary Perfusion Maps and Pulmonary Parenchyma Characteristics for the Semiautomated Classification of Pulmonary Hypertension
Source: Front Cardiovasc Med. 2022 Feb 28;9:835732. doi: 10.3389/fcvm.2022.835732 (PMC8982082; doi:10.3389/fcvm.2022.835732)
Supplement: Supplementary file 3 [file Data_Sheet_1.pdf]

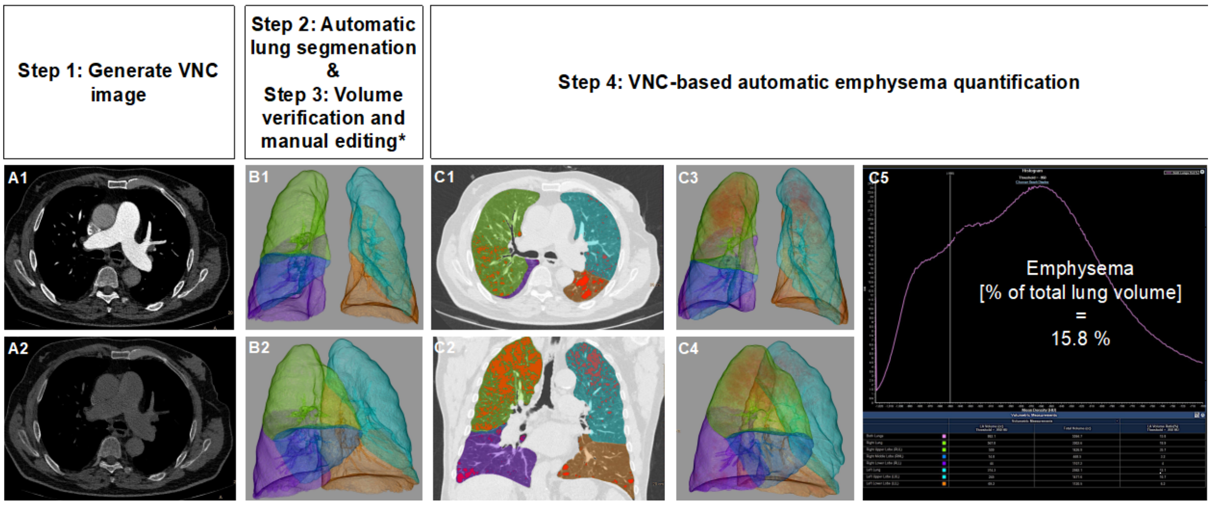

Supplementary Figure 1: Workflow of lung emphysema quantification.

Step 1 (Panel A1 and A2): Spectral reconstruction of VNC image. Step 2 and Step 3 (Panel B1 and B2): Automatic lung segmentation, volume verification and in case of insufficient segmentation manual editing (see Figure 2). Step 4 (Panel C1-C5): VNC-based automatic emphysema quantification (emphysematous lung areas marked in red) via thresholding, applying a threshold of -950 Hounsfield units (vertical white line Panel C5).

VNC, virtual non-contrast.
